# Association of Healthy Lifestyle and Life Expectancy in Patients With Cardiometabolic Multimorbidity: A Prospective Cohort Study of UK Biobank

**DOI:** 10.3389/fcvm.2022.830319

**Published:** 2022-06-09

**Authors:** Xunjie Cheng, Feiyun Ouyang, Tianqi Ma, Yi Luo, Jinghua Yin, Jinchen Li, Guogang Zhang, Yongping Bai

**Affiliations:** ^1^Department of Geriatric Medicine, Center of Coronary Circulation, Xiangya Hospital, Central South University, Changsha, China; ^2^National Clinical Research Center for Geriatric Disorders, Xiangya Hospital, Central South University, Changsha, China; ^3^Department of Social Medicine and Health Management, Central South University, Changsha, China; ^4^Department of Geriatric Medicine, Xiangya Hospital, Central South University, Changsha, China; ^5^Department of Cardiovascular Medicine, Xiangya Hospital, Central South University, Changsha, China; ^6^Department of Cardiovascular Medicine, The Third Xiangya Hospital, Central South University, Changsha, China

**Keywords:** multimorbidity, healthy lifestyle, life expectancy, mortality, cardiometabolic

## Abstract

**Background:**

The prevalence of cardiometabolic multimorbidity (CMM), which significantly increases the risk of mortality, is increasing globally. However, the role of healthy lifestyle in the secondary prevention of CMM is unclear.

**Methods:**

In total, 290,795 participants with CMM, which was defined as coexistence of at least two of hypertension (HTN), diabetes mellitus (DM), coronary heart disease (CHD), and stroke (ST), and those without these four diseases at baseline were derived from UK Biobank. The associations between specific CMM patterns and mortality, and that between healthy lifestyle (including physical activity, smoking, alcohol consumption, and vegetable and fruit consumption) and mortality in patients with specific CMM patterns were calculated using the flexible parametric Royston-Parmar proportion-hazard model. Hazard ratios (HRs) and corresponding 95% confidence intervals (CIs) were calculated.

**Results:**

During a median 12.3-year follow up period, 15,537 (5.3%) deaths occurred. Compared with participants without cardiometabolic diseases, the HRs for all-cause mortality were 1.54 [95% confidence interval (CI): 1.30, 1.82] in participants with HTN + DM, 1.84 (95% CI: 1.59, 2.12) in those with HTN + CHD, 1.89 (95% CI: 1.46, 2.45) in those with HTN + ST, and 2.89 (95% CI: 2.28, 3.67) in those with HTN + DM + CHD. At the age of 45 years, non-current smoking was associated with an increase in life expectancy by 3.72, 6.95, 6.75, and 4.86 years for participants with HTN + DM, HTN + CHD, HTN + ST, and HTN + DM + CHD, respectively. A corresponding increase by 2.03, 1.95, 2.99, and 1.88 years, respectively, was observed in participants with regular physical activity. Non-/moderate alcohol consumption and adequate fruit/vegetable consumption were not significantly associated with life expectancy in patients with specific CMM patterns.

**Conclusion:**

Cardiometabolic multimorbidity was associated with an increased risk of mortality. Regular physical activity and non-current smoking can increase life expectancy in patients with specific CMM patterns.

## Introduction

The number of patients living with cardiometabolic multimorbidity (CMM) has increased rapidly in recent decades due to population aging and the development of medical technology (1–3). A recent study on UK Biobank (UKB) participants showed that approximately 64, 59, 57, and 54% of patients with diabetes mellitus (DM), angina, stroke (ST), and myocardial infarction (MI) were also diagnosed with hypertension (HTN), respectively (2). Furthermore, CMM is associated with poor health outcomes (4–6). The co-existence of DM, MI, and ST was associated with a 3–5 times higher risk of mortality in those with CMM, compared with those who have none of these diseases (4). Despite the high prevalence of CMM and its poor prognosis, there is a paucity of studies reporting evidence for the primary and secondary prevention of CMM (7).

Healthy lifestyle, such as regular participation in physical activity and non-current smoking, is considered as cost-effective intervention to prevent the incidence of specific cardiometabolic disease and to improve the prognosis of patients with single cardiometabolic disease (8–13). However, to our knowledge, only four studies have explored the association between healthy lifestyle and mortality in patients with CMM, and the results varied widely in these studies (14–17). This could possibly be explained by the following reasons: (1) previous studies have used inconsistent CMM definitions. Although the incidence of CMM has been defined as the co-existence of at least two cardiometabolic diseases, different combinations of cardiometabolic diseases, such as DM, heart failure, peripheral vascular disease, and ST, have been used in previous studies (14–17). (2) Some studies had limited statistical power, with sample sizes ranging from 511 to 14,164 (14–17), and there were fewer than 20 events in some subgroups (14). (3) Studies that involved participants from different countries (the United Kingdom and China) (14–17). The different genetic, cultural backgrounds, and living environments may also influence the conclusion.

To further clarify the association between healthy lifestyle and mortality in patients with CMM and to provide evidence for the secondary prevention of CMM, we conducted the present study based on more than 0.5 million UKB participations. The aim of this study was to analyze whether and to what extent healthy lifestyle can increase life expectancy in patients with specific CMM patterns.

## Materials and Methods

### Study Population

The data used in this study were derived from the UKB project (Approved ID: 76118). Detailed information regarding the UKB has been well described in previous studies (18, 19). Briefly, more than 0.5 million participants from 22 assessment centers across England, Wales, and Scotland were recruited between 2006 and 2010. Demographic, clinical, and behavioral information were collected at baseline for all participants. Data on health outcomes, including new-onset diseases and deaths, were recorded during the follow-up. Written informed consent was obtained from each participant, and the UKB was approved by the National Health Service National Research Ethics Service.

In total, 502,414 participants were recruited. In this study, participants who died within 2 years of enrollment were excluded to minimize reverse causality (2,589). Participants with only one cardiometabolic disease (124,325) and participants with missing data on the main variables, including physical activity (72,610), smoking status (2,155), alcohol consumption (1,127), and vegetable and fruit consumption (9,324), were also excluded from the main analysis. Further, we excluded participants with missing information on other variables involved in our analyses (5,260). CMM patterns reported in less than 1,000 participants were also excluded to avoid unstable results from the small sample size. Finally, 290,795 participants were included in this study. Detailed information is provided in Supplementary Figure 1.

### Ascertainment of Cardiometabolic Multimorbidity

Different types of cardiometabolic diseases, such as HTN, DM, MI, and heart failure, have been used to define CMM in previous studies (4, 14, 20–22). To date, there is no consensus on the definition of CMM. According to previous studies and the findings reported in the UKB study (2), CMM was defined as the presence of at least two diseases of HTN, DM, coronary heart disease (CHD), and ST at baseline in this study. The incidence of CHD was defined as the presence of at least one out of MI and angina in this study. Six different sources of information were used to define the presence of these diseases, including self-reported non-cancer illness, diseases diagnosed by doctor, medication use, operation history, electronic health records coded by the International Classification of Diseases, ninth Revision (ICD-9), ICD-10, and Office of Population Censuses and Surveys Classification of Interventions and Procedures, version 4 (OPCS-4) (23, 24). Detailed information about the definition of these diseases is provided in Supplementary Table 1.

Theoretically, there are 11 different combinations of these four cardiometabolic diseases. To avoid unstable results from the small sample size, CMM patterns reported in <1,000 participants were excluded from this study. Finally, we categorized participants into the following five mutually exclusive patterns: (1) HTN + DM, (2) HTN + CHD, (3) HTN + ST, (4) HTN + DM + CHD, and (5) participants without cardiometabolic diseases.

### Ascertainment of Mortality

Data on deaths were obtained from the National Health Service Information Centre and the National Health Service Central Registers. The dates of death and the underlying causes of death based on the ICD-10 were recorded. The outcome of this study was all-cause mortality.

### Ascertainment of Main Variables

Four common lifestyle factors were assessed in this study: physical activity, smoking, alcohol consumption, and vegetable and fruit consumption. Information on these factors was collected using a touchscreen questionnaire at baseline.

(1) For physical activity, participants were asked “In a typical week, on how many days did you walk, do moderate physical activity, and do vigorous physical activity for at least 10 min at a time, respectively.” and “How many minutes did you usually spend walking, doing moderate physical activity, and doing vigorous physical activity on a typical DAY, respectively.” Regular physical activity was defined as engagement in at least 150 min of walking, moderate activity per week, or 75 min of vigorous activity, according to the 2019 UK Physical Activity guidelines (25). (2) For smoking status, self-reported smoking status, including current smoking, previous smoking, and never-smoking, was recorded. Participants were classified into current smoking and non-current smoking groups (including those in previous-smoking and never-smoking groups). (3) For alcohol consumption, participants were asked, “About how often do you drink alcohol?”. Participants were grouped into two alcohol consumption groups: non-/moderate alcohol consumption (<3 times per week) and excessive alcohol consumption (≥3 times per week). (4) Regarding vegetable and fruit consumption, participants were asked “About how many pieces of fresh or dried fruit would you eat per day?” and “On average, how many heaped tablespoons of cooked or salad/raw vegetables would you eat per day?”. The total portion of vegetable or fruit consumption was calculated as follows: a portion is one piece of fresh fruit, three pieces of dried fruit, four heaped tablespoons of salad/raw vegetables, and three heaped tablespoons of cooked vegetables. Participants were grouped into two diet groups: adequate fruit/vegetable consumption (≥5 portions of vegetables or fruits every day) and inadequate fruit/vegetable consumption [<5 portion(s) of vegetables or fruits every day] (26).

Several confounding variables were also included in this study: sex, race (white, non-white), body mass index [BMI, weight in kilograms (kg) divided by the square of the height in meters (m^2^)], socioeconomic status (Townsend deprivation index), sedentary time (time spent watching television, using computer, and driving), and use of antihypertensive medication, insulin, cholesterol lowering medication, and antiplatelet drugs (aspirin or clopidogrel).

### Statistics Analyses

Baseline characteristics of the five mutually exclusive groups were presented as median (interquartile range) for continuous variables (non-normal distribution) and number (percentage) for categorical variables. Using participants free of the four cardiometabolic diseases as reference, the association between specific CMM patterns and all-cause mortality was calculated using the flexible parametric Royston-Parmar proportion-hazard model with age as the time scale (27). Hazard ratios (HRs) and corresponding 95% confidence intervals (CIs) were calculated. Three different models were used for each CMM pattern. The first model (Model 1) was adjusted for gender and race. The second model (Model 2) was additionally adjusted for BMI and socioeconomic status. Model 3 was further adjusted for lifestyle factors, including sedentary time, physical activity, smoking status, alcohol consumption, and vegetable and fruit consumption, and use of antihypertensive medication, insulin, cholesterol lowering medication, and antiplatelet drugs.

To further explore whether and to what extent healthy lifestyle factors can improve the prognosis of patients with specific CMM patterns, the associations between lifestyle factors and mortality were calculated for each CMM pattern using a flexible parametric Royston-Parmar proportion-hazard model with age as the time scale (27). Unhealthy behaviors (e.g., physical inactivity, current smoking, excessive alcohol consumption, and inadequate fruit/vegetable consumption) were used as references in each analysis. We fitted three different model for each CMM pattern, respectively. Model 1 included only these four lifestyle factors, model 2 was additionally adjusted for gender and race, and model 3 was further adjusted for BMI, socioeconomic status, sedentary time, and use of antihypertensive medication, insulin, cholesterol lowering medication, and antiplatelet drugs. Associations between healthy lifestyle and life expectancy were also calculated in this study. For each lifestyle factor, we estimated the population-averaged survival curves at ages 45–100 years based on the assumption of whether they had a healthy lifestyle, after adjusting for all variables (14, 15). The restricted mean survival times (RMSTs), which is the area under the survival curve, were calculated, and increase of life expectancy was quantified as the difference in RMSTs (28).

### Sensitivity Analyses

We performed five sensitivity analyses to assess the robustness of the association between healthy lifestyle and life expectancy in patients with CMM. First, participants with missing data on daily duration and number of day(s)/week for physical activities were excluded from the main analysis. In the sensitivity analysis, only participants with missing data for number of day(s)/week were excluded, and the minimum duration (10) minutes of physical activity in each question, was used to replace the missing data. This resulted in 3,137 additional participants in the analysis. Second, participants were also asked, “In an average week, how many glasses of red wine, glasses of white wine, pints of beer or cider, measures of spirits or liqueurs, and glasses of fortified wine would you drink, respectively?”. The total amount of alcohol consumption was converted into standard units: 1.5 units for one glass of red or white wine, 2 units for one pint of beer or cider, 1.0 unit for a single spirits or liqueurs, and 1.0 unit for a small glass of fortified wine. Participants were grouped into two alcohol consumption groups based on the recommendation of the National Health Service: non-/moderate alcohol consumption (<14 units per week) and excessive alcohol consumption (≥14 units per week) (29). Amount, instead of frequency of alcohol consumption was used in the sensitivity analysis. Third, according to the American Heart Association, a more restricted healthy diet was defined as a balanced intake of fruit, vegetables, fish, processed foods, and red meat in the sensitivity analysis (30). Fourth, considering that participants with newly diagnosed cardiometabolic diseases (<1 year) may change their behaviors during follow-up, they were excluded from the sensitivity analysis (5,141 participants). Fifth, there were 2,429, 2,332, 776, and 436 new-onset cardiometabolic diseases during follow-up in participants with HTN + DM, HTN + CHD, HTN + ST, HTN + DM + CHD, respectively. These participants were excluded in the sensitivity analysis.

## Results

### Baseline Characteristics

In total, 290,795 participants with complete data were included in this study, and 263,396 (90.6%) of them were free of HTN, DM, CHD, and ST at baseline (Table 1). Among participants with CMM (27,399) at baseline, 10,286 (37.5%) had HTN + DM, 11,146 (40.7%) had HTN + CHD, 3,049 (11.1%) had HTN + ST, and 2,918 (10.7%) had HTN + DM + CHD. Compared with participants without these diseases, those with CMM were older, more likely to be males, and lived in deprived areas. There were also some differences in lifestyle factors between the participants with and without cardiometabolic diseases at baseline. For example, participants with HTN + DM were more likely to be physically inactive (82.7 vs. 74.2%), and participants without cardiometabolic diseases were more likely to be inadequate fruit/vegetable consumption (25.5 vs. 31.1%).

**TABLE 1:** Baseline characteristics of participants by disease status at baseline.

Characteristics	Non-CMD	HTN + DM	HTN + CHD	HTN + ST	HTN + DM + CHD
No. of participants	263,396	10,286	11,146	3,049	2,918
Age, median (IQR), year	55.8 (48.5, 62.1)	61.7 (56.0, 65.7)	63.9 (59.8, 67.0)	63.6 (58.4, 66.8)	63.7 (59.6, 67.0)
Male, No. (%)	114,577 (43.5)	6,378 (62.0)	8,115 (72.8)	1,814 (59.5)	2,228 (76.4)
White, No. (%)	251,635 (95.5)	9,133 (88.8)	10,694 (95.9)	2,952 (96.8)	2,591 (88.8)
Townsend Deprivation Index, median (IQR)	–2.31 (-3.73, 0.13)	–1.53 (-3.31, 1.64)	–1.91 (-3.50, 1.08)	–1.95 (–3.50, 0.95)	–0.91 (-3.13, 2.53)
BMI, median (IQR)	25.9 (23.54, 28.69)	31.11 (27.87, 35.13)	28.47 (25.93, 31.58)	27.96 (25.48, 31.14)	31.03 (27.94, 34.84)
Sedentary time, median (IQR), hour	4 (3, 6)	5 (4, 7)	5 (4, 6.5)	5 (4, 6.5)	5.5 (4, 7.5)
Regular physical activity, No. (%)	217,785 (82.7)	7,630 (74.2)	8,915 (80.0)	2,350 (77.1)	2,059 (70.6)
None/moderate alcohol consumption, No. (%)	142,406 (54.1)	6,860 (66.7)	6,097 (54.7)	1,745 (57.2)	2,021 (69.3)
Adequate fruit/vegetable consumption, No. (%)	67,261 (25.5)	3,197 (31.1)	2,926 (26.3)	835 (27.4)	887 (30.4)
Non-current smoking, No. (%)	236,418 (89.8)	9,325 (90.7)	9,952 (89.3)	2,682 (88.0)	2,608 (89.4)
Antihypertensive use, No. (%)	0 (0.0)	8,949 (87.0)	9,742 (87.4)	2,571 (84.3)	2,645 (90.6)
Cholesterol lowering medication use, No. (%)	13,409 (5.1)	7,870 (76.5)	9,387 (84.2)	2,086 (68.4)	2,666 (91.4)
Insulin use, No. (%)	0 (0.0)	1,924 (18.7)	0 (0.0)	0 (0.0)	672 (23.0)
Antiplatelet use, No. (%)	13,672 (5.2)	5,069 (49.3)	9,099 (81.6)	2,052 (67.3)	2,488 (85.3)

*Non-CMD, participants without hypertension, diabetes mellitus, coronary heart disease, and stroke; HTN, hypertension; DM, diabetes mellitus; CHD, coronary heart disease; ST, stroke; IQR, interquartile range; BMI, body mass index. Regular physical activity means at least 150 min of walking, moderate activity per week, or 75 min of vigorous activity. None/moderate alcohol consumption means <3 times per week. Adequate fruit/vegetable consumption means ≥5 portions every day.*

### CMM and Mortality

In total, 15,537 deaths occurred during a median follow-up of 12.3 years, and the numbers of deaths were 11,214, 1,426, 1,658, 487, and 752 in participants with no cardiometabolic diseases, HTN + DM, HTN + CHD, HTN + ST, HTN + DM + CHD, respectively.

Compared with participants free of these diseases at baseline, the prevalence of CMM was associated with an increased risk of mortality. In the fully adjusted models (Table 2), the risk of mortality increased by 54% (HR: 1.54, 95% CI: 1.30, 1.82) for participants with HTN + DM, 84% (HR: 1.84, 95% CI: 1.59, 2.12) for those with HTN + CHD, 89% (HR: 1.89, 95% CI: 1.46, 2.45) for those with HTN + ST, and 189% (HR: 2.89, 95% CI: 2.28, 3.67) for those with HTN + DM + CHD.

**TABLE 2:** Association of specific cardiometabolic multimorbidity with all-cause mortality.

CMM pattern	Model 1		Model 2		Model 3	
	HR (95% CI)	*P*	HR (95% CI)	*P*	HR (95% CI)	*P*
HTN + DM	2.13 (2.01, 2.25)	<0.001	1.85 (1.74, 1.96)	<0.001	1.54 (1.30, 1.82)	<0.001
HTN + CHD	1.81 (1.72, 1.91)	<0.001	1.68 (1.59, 1.77)	<0.001	1.84 (1.59, 2.12)	<0.001
HTN + ST	2.13 (1.94, 2.33)	<0.001	2.00 (1.82, 2.19)	<0.001	1.89 (1.46, 2.45)	<0.001
HTN + DM + CHD	3.38 (3.14, 3.65)	<0.001	2.86 (2.64, 3.09)	<0.001	2.89 (2.28, 3.67)	<0.001

*CMM, cardiometabolic multimorbidity; HTN, hypertension; DM, diabetes mellitus; CHD, coronary heart disease; ST, stroke; HR, hazard ratio; CI, confidence interval. Model 1: adjusted for gender and race. Model 2: additionally adjusted for body mass index and Townsend deprivation index. Model 3: further adjusted for sedentary time, physical activity, smoking status, alcohol consumption, and fruit/vegetable consumption.*

### Healthy Lifestyle and Mortality in Patients With CMM

**FIGURE 1:**
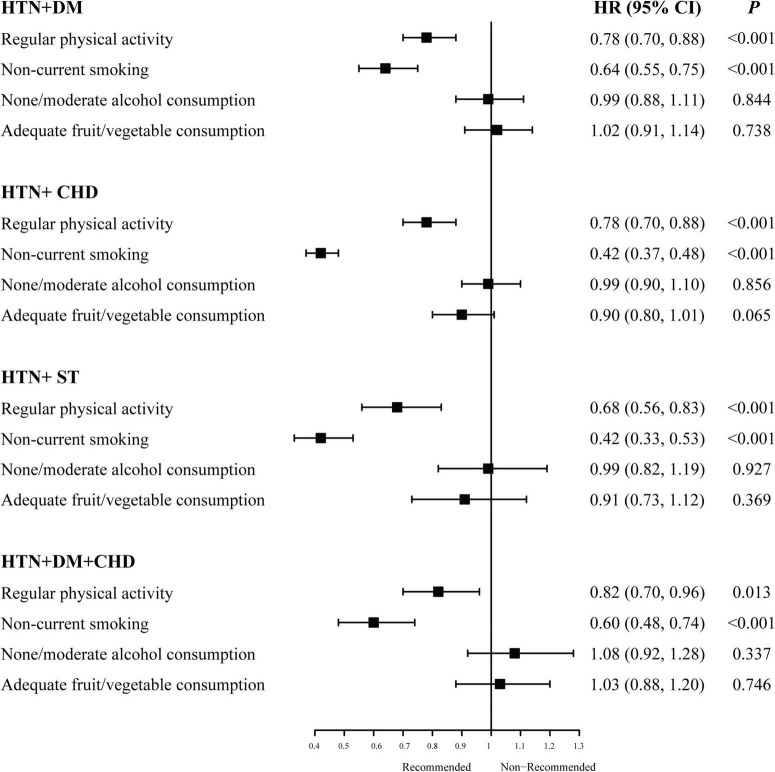
Association of healthy lifestyle and all-cause mortality in specific cardiometabolic multimorbidity patterns. HTN, hypertension; DM, diabetes mellitus; CHD, coronary heart disease; ST, stroke; HR, hazard ratio; CI, confidence interval. HRs here were adjusted for gender, race, body mass index, Townsend deprivation index, sedentary time and the other three healthy lifestyle factors for each healthy lifestyle factor.

Figure 1 shows that the association between lifestyle factors and mortality was similar among patients with different CMM patterns. Regular physical activity and non-current smoking were associated with a decreased risk of mortality in all four CMM patterns, and the association of non-/moderate alcohol consumption or adequate fruit/vegetable consumption with all-cause mortality was not statistically significant in any of the four CMM patterns. Adequate fruit/vegetable consumption showed a marginal protective role in patients with HTN + CHD (HR: 0.90, 95% CI: 0.80, 1.01).

Among the different patterns, non-current smoking significantly decreased the risk of mortality by 58% (HR: 0.42, 95% CI: 0.37, 0.48) in patients with HTN + CHD and HTN + ST (HR: 0.42, 95% CI: 0.33, 0.53). Engagement in regular physical activity significantly decreased mortality risk in patients with HTN + ST (HR: 0.68, 95% CI: 0.56, 0.83), and the HRs were similar in the other groups (HRs: 0.78∼0.82). The detailed results are provided in Supplementary Table 2.

### Healthy Lifestyle and Life Expectancy in Patients With CMM

**FIGURE 2:**
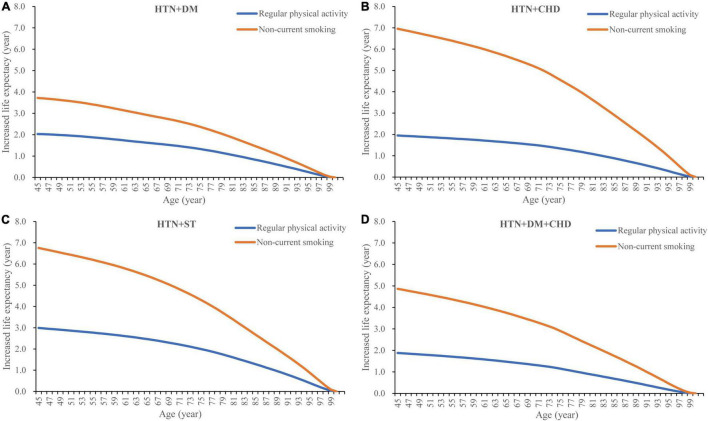
Healthy lifestyle and life expectancy in specific cardiometabolic multimorbidity patterns. HTN, hypertension; DM, diabetes mellitus; CHD, coronary heart disease; ST, stroke.

Details regarding the association between healthy lifestyle and life expectancy are shown in Figure 2 and Supplementary Table 3. The findings showed that non-current smoking was associated with a significant increase in life expectancy in all four CMM patterns. At the age of 45 years, non-current smoking was associated with an increase life expectancy by 3.72, 6.95, 6.75, and 4.86 years in participants with HTN + DM, HTN + CHD, HTN + ST, and HTN + DM + CHD, respectively. Life expectancy increased by 2.93, 5.67, 5.44, and 3.75 years in participants aged 65 years, respectively. Regular engagement in physical activity was also associated with an increase in life expectancy (ranging from 1.88 to 2.99 years at age 45 years, and from 1.48 to 2.47 years at age 60 years) in all four CMM patterns.

### Sensitivity Analyses

The results of the sensitivity analyses were mostly consistent with the findings of the main analysis, except for vegetable and fruit consumption. Consumption of a more restricted healthy diet decreased the risk of mortality in patients with HTN + CHD (HR: 0.82, 95% CI: 0.74, 0.90). Results from the other sensitivity analyses also showed that adequate fruit/vegetable consumption may improve prognosis in patients with HTN + CHD, even they were not all statistically significant (*P* = 0.187, 0.097, 0.039, and 0.121, respectively). Detailed results of the sensitivity analysis are provided in Supplementary Tables 4–8.

## Discussion

### Principal Findings

Based on the data of approximately half a million participants from the UKB study, we analyzed the association of specific CMM patterns with mortality and estimated whether and to what extent healthy lifestyles increase life expectancy in patients with specific CMM patterns. There were three main findings: (1) The presence of CMM was associated with an increased risk of mortality compared with the participants without cardiometabolic diseases; (2) non-current smoking and regular physical activity were associated with a decreased risk of mortality and increased life expectancy in patients with specific CMM patterns; and (3) adequate fruit/vegetable consumption and non-/moderate alcohol consumption were not significantly associated with mortality in patients with specific CMM patterns in this study. These findings have potential implications for health researchers and policymakers in planning more targeted lifestyle interventions.

### Comparison With Other Studies

In this study, we found that CMM significantly increased the risk of mortality. This was consistent with the results of previous studies that indicated that patients with more than one of HTN, DM, MI, and ST had 1.7 to 6.0 times higher risk of mortality than patients without these diseases (4, 5). The HRs (1.54–1.89) were similar in patients with two cardiometabolic diseases, but increased greatly (2.89) in patients with three cardiometabolic diseases (HTN + DM + CHD), which was also consistent with findings from previous studies (4, 5). These results hinted that cost-effective strategies should be implemented to prevent the incidence of CMM. Beyond medication treatment, lifestyle intervention is the most-commonly recommended strategy. Although it is well studied that some modifiable lifestyle factors, such as non-current smoking and regular physical activity, can decrease the incidence of specific cardiometabolic diseases in the general population or the risk of mortality in patients with a single cardiometabolic disease (9, 11, 12), some other studies have shown that these factors may play different roles in the progression from single disease to multimorbidity (16, 31). Results from a study indicated that physical activity cannot prevent the incidence of multimorbidity in patients with cancer (95% CI: 0.93, 1.07) and cardiovascular disease (95% CI: 0.92, 1.05) (31). Therefore, more studies are required to clarify the progress of CMM in patients with a single cardiometabolic disease and to explore cost-effective lifestyle intervention strategies.

To our knowledge, only four previous studies have explored the association between healthy lifestyle and mortality in patients with CMM (14–17). However, the results were inconsistent in the four studies and this study. Two previous studies explored the association between four lifestyle factors and mortality in participants with CMM in the United Kingdom: Chudasama et al. indicated that non-current smoking was not associated with mortality (HR: 0.78, 95% CI: 0.57, 1.06), but Singh-Manoux et al. showed that current smoking can significantly increase mortality risk (HR: 1.59, 95% CI: 1.12, 2.26) (14, 16). In this study, we found that non-current smoking was associated with decreased mortality risk in all four CMM patterns. Han et al. found that tobacco smoking was associated with an increased risk of mortality (HR: 1.27, 95% CI: 1.16, 1.40) in Chinese population with two or more of the following cardiometabolic diseases: type 2 diabetes, ischemic heart disease, and ST (17). As mentioned before, these inconsistent results were possibly caused by the different definitions of CMM, limited statistical power, and population heterogeneity. In contrast to previous studies, we explored the association between healthy lifestyle and mortality in patients with specific CMM patterns, and CMM patterns reported in less than 1,000 patients were excluded to maintain stable results. Despite these advantages, the high heterogeneity of results from different studies indicates that more research is required to further clarify the potential beneficial effects of healthy lifestyle in patients with CMM.

The results of this study indicated that regular physical activity and non-current smoking, rather than non-/moderate alcohol consumption and adequate fruit/vegetable consumption, can increase life expectancy in patients with CMM. In line with our findings, previous studies found that alcohol consumption was associated with poor health outcomes, and that an adequate fruit/vegetable consumption was not associated with lower mortality risk in older people with more than two chronic diseases (32–34). Although some previous studies found that moderate alcohol consumption was associated with decreased risk of some diseases, such as DM and obesity, a recent study indicated that this may be misleading due to misreporting and longitudinal changes in alcohol consumption (34). In the present study, non-current smoking was associated with increased life expectancy (3.72–6.95 years at age 45 years) in all four CMM patterns, followed by engagement in physical activity. This was consistent with findings of previous studies showing that irregular physical activity and smoking were associated with increased mortality risk in both the general population and those with cardiometabolic disease (9, 10). Despite the adverse effects of smoking on health outcomes, the prevalence of smoking remains very high in some countries. In 2015, a study indicated that smoking prevalence was approximately 25.0 and 5.4% in men and women worldwide, respectively (35). The prevalence was much higher and stable in Chinese male patients, approximately 48.4% in 2003, 47.0% in 2008, and 47.2% in 2013 (36). To decrease the rate of premature mortality caused by smoking and to improve healthy life expectancy, some effective interventions, such as smoking bans, health warnings, advertising bans, and tobacco taxes, should be implemented to prevent smoking-related disease burden (37).

### Policy Implications

There are several intermediate phases from healthy to death states. The role of healthy lifestyle in three common transitions: (1) healthy to one disease, (2) healthy to death, and (3) one disease to death, have been well studied in previous studies. However, the number of patients living with multimorbidity has increased dramatically in recent years because of population aging and medical advances, and whether and to what extent a healthy lifestyle can modify the transition from one disease to multimorbidity and succedent death, has been unclear. To our knowledge, this is the first study to explore the role of healthy lifestyle in the secondary prevention of specific CMM patterns. We found that an adequate fruit/vegetable consumption was not associated with decreased mortality risk in all four CMM patterns. This was different from previous findings that reported a positive effect of adequate fruit/vegetable consumption on decreased mortality risk (HR: 0.95, 95% CI: 0.92–0.98) in the general population (13). These inconsistent results further indicated that the beneficial effects of some modifiable lifestyle factors may change between health transitions (16, 17). Consequently, more studies are required to explore cost-effective primary and secondary prevention strategies for CMM.

### Strengths and Limitations

This study has several strengths. First, the UKB is a large-scale, population-based prospective cohort study; hence, the present study has statistical power. Second, CMM was classified into four specific patterns, this avoided the inconsistent definitions used in previous studies. Lastly, beyond hazard ratios, this study also reported an association between healthy lifestyle and life expectancy, which is a more understandable indicator in the general population.

This study also has some limitations. First, reverse causality may exist in our study, although we excluded participants who died during the first two years of follow-up from the main analysis, and the results remained unchanged when participants with newly diagnosed (<1 year) cardiometabolic disease were excluded from the sensitivity analysis. Second, only four cardiometabolic diseases were included in this study; some other diseases, such as cancer and depression, were not considered. Too many diseases will result in many specific multimorbidity patterns (26 for five diseases and 57 for six diseases); moreover, these four cardiometabolic diseases share a similar pathogenesis. Third, all variables used in this study were collected at baseline and assumed to be unchanged during the follow-up, and further studies with repeated measurements of these variables are necessary to verify our results. Finally, the population sample of the UKB study are not entirely representative; hence, the results from the present study should be carefully generalized to other populations.

## Conclusion

In conclusion, our findings showed that regular physical activity and non-current smoking, rather than non-/moderate alcohol consumption and adequate fruit/vegetable consumption, decreased mortality risk and increased life expectancy in patients with specific CMM patterns. These results proved the importance of healthy lifestyle in the secondary prevention of CMM. Further studies are required to verify these results in other populations.

## Data Availability Statement

Data was obtained from UK Biobank and are available at https://www.ukbiobank.ac.uk/ with the permission of UK Biobank.

## Ethics Statement

The studies involving human participants were reviewed and approved by National Health Service National Research Ethics Service. The patients/participants provided their written informed consent to participate in this study.

## Author Contributions

XC and YB designed the study. XC wrote the manuscript. FO, TM, YL, and JY contributed to the data analysis and data interpretation. JL, GZ, and YB contributed to the revision of the manuscript. All authors contributed to the article and approved the submitted version.

## Conflict of Interest

The authors declare that the research was conducted in the absence of any commercial or financial relationships that could be construed as a potential conflict of interest.

## Publisher’s Note

All claims expressed in this article are solely those of the authors and do not necessarily represent those of their affiliated organizations, or those of the publisher, the editors and the reviewers. Any product that may be evaluated in this article, or claim that may be made by its manufacturer, is not guaranteed or endorsed by the publisher.
