# Supplementary material for: Association of Healthy Lifestyle and Life Expectancy in Patients With Cardiometabolic Multimorbidity: A Prospective Cohort Study of UK Biobank
Source: Front Cardiovasc Med. 2022 Jun 9;9:830319. doi: 10.3389/fcvm.2022.830319 (PMC9218816; doi:10.3389/fcvm.2022.830319)
Supplement: Supplementary file 1 [file Data_Sheet_1.docx]

Supplementary Material

# Supplementary Figures and Tables

## Supplementary Figures


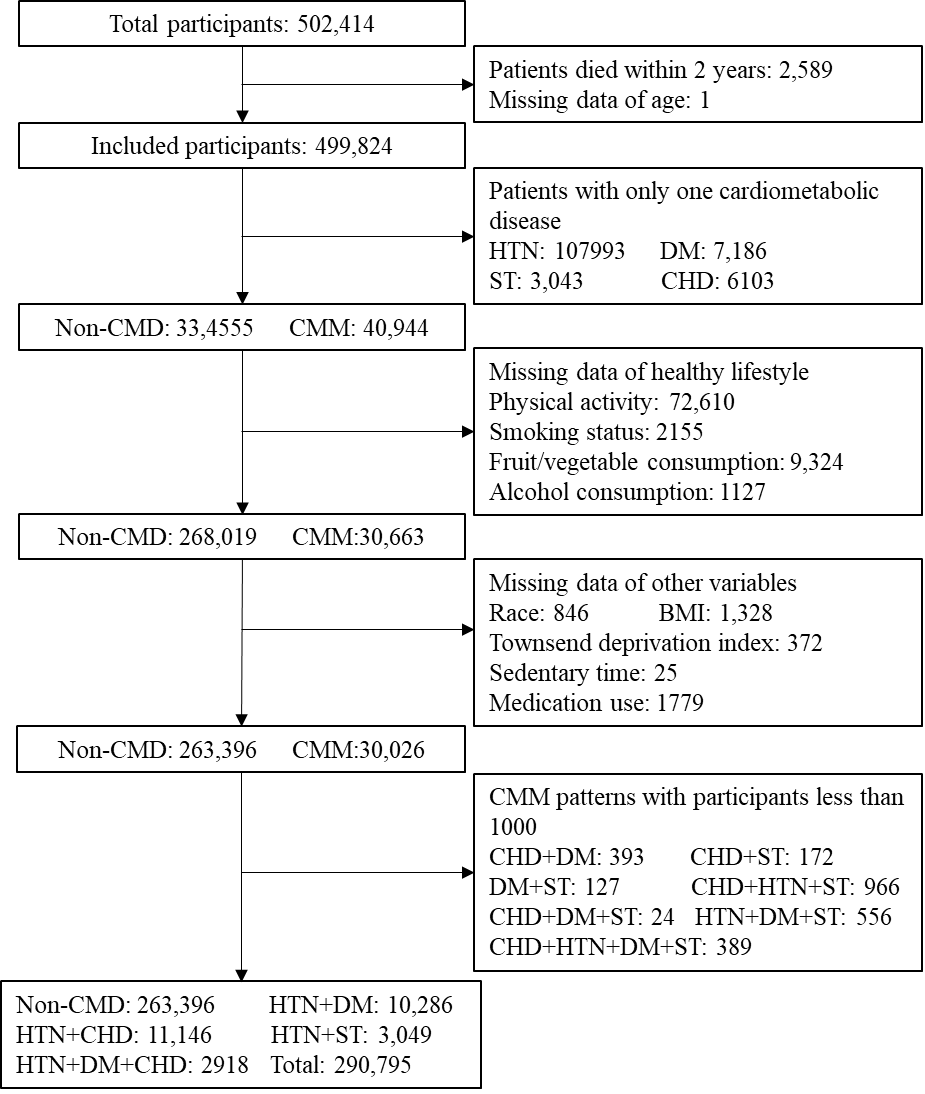


**Supplementary Figure 1.** Study flowchart

CHD means coronary heart disease. HTN means hypertension. DM means diabetes mellitus. ST means stroke. Non-CMD means participants free of these four cardiometabolic diseases.

## Supplementary Tables

**Supplementary Table 1**. Cardiometabolic diseases definitions used in UK Biobank

| Disease | ICD-9 | ICD-10 | OPCS-4 | Self-reported fields |
| --- | --- | --- | --- | --- |
| Coronary heart disease | 41271 (410-414) | 41270 (I20-I25, Z95.1, Z95.5), 131296, 131298, 131300, 131302, 131304, 131306 | 41272 (K40-K46, K49, K50, K75) | 6150 (1, 2), 3894, 3627, 20002 (1074, 1075), 20004 (1070,1095,1523) |
| Hypertension | 41271 (401-405) | 41270 (I10-I13, I15, O10), 131286, 131288, 131290, 131292, 131294, 132180 |  | 6150 (4), 6153 (2), 6177 (2), 2966, 20002 (1065, 1072) |
| Diabetes mellitus | 41271 (250, 3572, 3620) | 41270 (E10-E14, G59.0, G63.2, H28.0, H36.0, M14.2, N08.3), 130706, 130708, 130710, 130712, 130714 |  | 6153 (3), 6177 (3), 2443 (1), 2976, 20002 (1220, 1222, 1223) |
| Stroke | 41271 (3361, 3623, 430, 431, 4329, 4330, 4331, 4332, 4333, 4338, 4339, 434, 436) | 41270 (I60, I61, I62.9, I63, I64, I67.8, I69.0, I69.3, G95.1, H34.1, H34.2, S06.6), 131180, 131360, 131362, 131364, 131368, 131370, 131372, 131374, 131376, 131378 | 41272 (A05.2-A05.4, L35.1, L35.3, L34.3) | 6150 (3), 4056, 20002 (1081, 1491, 1583, 1086) |

ICD means International Classification of Diseases. OPCS-4 means Office of Population Censuses and Surveys Classification of Interventions and Procedures, version 4.

**Supplementary Table 2**. Association of healthy lifestyle and mortality in participants with cardiometabolic multimorbidity

| Cardiometabolic multimorbidity | Model 1 | |  | Model 2 | |  | Model 3 | |
| --- | --- | --- | --- | --- | --- | --- | --- | --- |
|  | HR (95% CI) | *P* |  | HR (95% CI) | *P* |  | HR (95% CI) | *P* |
| **HTN+DM** |  |  |  |  |  |  |  |  |
| Regular physical activity | 0.73 (0.65, 0.81) | **<0.001** |  | 0.73 (0.65, 0.82) | **<0.001** |  | 0.78 (0.70, 0.88) | **<0.001** |
| Non-current smoking | 0.59 (0.50, 0.69) | **<0.001** |  | 0.60 (0.52, 0.70) | **<0.001** |  | 0.64 (0.55, 0.75) | **<0.001** |
| None/moderate alcohol consumption | 0.98 (0.87, 1.09) | 0.666 |  | 1.09 (0.98, 1.22) | 0.126 |  | 0.99 (0.88, 1.11) | 0.844 |
| Adequate fruit/vegetable consumption | 0.99 (0.89, 1.11) | 0.896 |  | 1.03 (0.92, 1.16) | 0.591 |  | 1.02 (0.91, 1.14) | 0.738 |
| **HTN+CHD** |  |  |  |  |  |  |  |  |
| Regular physical activity | 0.75 (0.67, 0.84) | **<0.001** |  | 0.75 (0.67, 0.84) | **<0.001** |  | 0.78 (0.70, 0.88) | **<0.001** |
| Non-current smoking | 0.38 (0.34, 0.44) | **<0.001** |  | 0.39 (0.34, 0.44) | **<0.001** |  | 0.42 (0.37, 0.48) | **<0.001** |
| None/moderate alcohol consumption | 0.96 (0.88, 1.06) | 0.464 |  | 1.04 (0.94, 1.15) | 0.433 |  | 0.99 (0.90, 1.10) | 0.856 |
| Adequate fruit/vegetable consumption | 0.87 (0.77, 0.97) | **0.015** |  | 0.90 (0.80, 1.01) | 0.078 |  | 0.90 (0.80, 1.01) | 0.065 |
| **HTN+ST** |  |  |  |  |  |  |  |  |
| Regular physical activity | 0.66 (0.54, 0.80) | **<0.001** |  | 0.65 (0.53, 0.79) | **<0.001** |  | 0.68 (0.56, 0.83) | **<0.001** |
| Non-current smoking | 0.36 (0.29, 0.45) | **<0.001** |  | 0.36 (0.29, 0.45) | **<0.001** |  | 0.42 (0.33, 0.53) | **<0.001** |
| None/moderate alcohol consumption | 0.94 (0.78, 1.13) | 0.495 |  | 1.02 (0.85, 1.23) | 0.810 |  | 0.99 (0.82, 1.19) | 0.927 |
| Adequate fruit/vegetable consumption | 0.85 (0.69, 1.04) | 0.116 |  | 0.90 (0.73, 1.11) | 0.322 |  | 0.91 (0.73, 1.12) | 0.369 |
| **HTN+DM+CHD** |  |  |  |  |  |  |  |  |
| Regular physical activity | 0.74 (0.64, 0.86) | **<0.001** |  | 0.74 (0.63, 0.86) | **<0.001** |  | 0.82 (0.70, 0.96) | **0.013** |
| Non-current smoking | 0.53 (0.43, 0.66) | **<0.001** |  | 0.55 (0.44, 0.67) | **<0.001** |  | 0.60 (0.48, 0.74) | **<0.001** |
| None/moderate alcohol consumption | 1.14 (0.97, 1.34) | 0.105 |  | 1.23 (1.05, 1.45) | **0.010** |  | 1.08 (0.92, 1.28) | 0.337 |
| Adequate fruit/vegetable consumption | 1.02 (0.87, 1.19) | 0.799 |  | 1.06 (0.91, 1.24) | 0.436 |  | 1.03 (0.88, 1.20) | 0.746 |

CHD means coronary heart disease. HTN means hypertension. DM means diabetes mellitus. ST means stroke, HR means hazard ratio, CI means confidence interval.

Physical inactivity, current smoking, excess alcohol consumption and inadequate fruit/vegetable consumption were used as reference groups. Model 1 was unadjusted; model 2 was adjusted for gender and race; model 3 was further adjusted for body mass index, Townsend deprivation index and sedentary time, and use of antihypertensive medication, insulin, cholesterol lowering medication, and antiplatelet drugs.

**Supplementary Table 3**. Increased life expectancy by regular physical activity and non-current smoking at different ages

| Cardiometabolic multimorbidity | Age at 45 years (95% CI) | Age at 65 years (95% CI) | Age at 85 years (95% CI) |
| --- | --- | --- | --- |
| **HTN+DM** |  |  |  |
| Regular physical activity | 2.03 (1.02, 3.05) | 1.63 (0.82, 2.44) | 0.84 (0.41, 1.27) |
| Non-current smoking | 3.72 (2.25, 5.19) | 2.93 (1.78, 4.09) | 1.60 (0.96, 2.23) |
| **HTN+CHD** |  |  |  |
| Regular physical activity | 1.95 (0.98, 2.92) | 1.63 (0.82, 2.44) | 0.88 (0.43, 1.32) |
| Non-current smoking | 6.95 (5.44, 8.47) | 5.67 (4.46, 6.89) | 2.91 (2.12, 3.69) |
| **HTN+ST** |  |  |  |
| Regular physical activity | 2.99 (1.23, 4.75) | 2.47 (1.01, 3.92) | 1.29 (0.47, 2.10) |
| Non-current smoking | 6.75 (4.19, 9.32) | 5.44 (3.36, 7.53) | 2.68 (1.39, 3.97) |
| **HTN+DM+CHD** |  |  |  |
| Regular physical activity | 1.88 (0.34, 3.43) | 1.48 (0.28, 2.68) | 0.68 (0.12, 1.25) |
| Non-current smoking | 4.86 (2.64, 7.09) | 3.75 (2.08, 5.43) | 1.74 (0.93, 2.55) |

CHD means coronary heart disease. HTN means hypertension. DM means diabetes mellitus. ST means stroke, CI means confidence interval.

**Supplementary Table 4.** Sensitivity analysis results after imputing missing physical activity data

| Cardiometabolic multimorbidity | Number of  participants | Number of  deaths | HR (95% CI) | *P* | Increased life expectancy (95% CI) | |
| --- | --- | --- | --- | --- | --- | --- |
|  |  |  |  |  | Age at 45 years | Age at 65 years |
| **HTN+DM** |  |  |  |  |  |  |
| Regular physical activity | 12348 | 1719 | 0.79 (0.71, 0.88) | <0.001 | 1.89 (1.01, 2.77) | 1.52 (0.81, 2.22) |
| Non-current smoking |  |  | 0.60 (0.52, 0.69) | <0.001 | 4.16 (2.82, 5.50) | 3.27 (2.23, 4.32) |
| None/moderate alcohol consumption |  |  | 1.00 (0.90, 1.11) | 0.966 | / | / |
| Adequate fruit/vegetable consumption |  |  | 1.04 (0.93, 1.15) | 0.500 | / | / |
| **HTN+CHD** |  |  |  |  |  |  |
| Regular physical activity | 13343 | 2000 | 0.79 (0.71, 0.87) | <0.001 | 1.89 (1.04, 2.73) | 1.58 (0.87, 2.78) |
| Non-current smoking |  |  | 0.41 (0.37, 0.46) | <0.001 | 7.16 (5.77, 8.56) | 5.83 (4.71, 6.95) |
| None/moderate alcohol consumption |  |  | 0.94 (0.86, 1.03) | 0.193 | / | / |
| Adequate fruit/vegetable consumption |  |  | 0.93 (0.84, 1.04) | 0.187 | / | / |
| **HTN+ST** |  |  |  |  |  |  |
| Regular physical activity | 3695 | 584 | 0.69 (0.57, 0.82) | <0.001 | 2.95 (1.42, 4.49) | 2.45 (1.19, 3.72) |
| Non-current smoking |  |  | 0.39 (0.32, 0.49) | <0.001 | 7.37 (5.02, 9.72) | 5.97 (4.09, 7.85) |
| None/moderate alcohol consumption |  |  | 0.97 (0.82, 1.15) | 0.733 | / | / |
| Adequate fruit/vegetable consumption |  |  | 0.92 (0.75, 1.11) | 0.370 | / | / |
| **HTN+DM+CHD** |  |  |  |  |  |  |
| Regular physical activity | 3477 | 895 | 0.82 (0.71, 0.95) | 0.006 | 1.95 (0.51, 3.39) | 1.51 (0.40, 2.61) |
| Non-current smoking |  |  | 0.63 (0.52, 0.76) | <0.001 | 4.61 (2.51, 6.72) | 3.51 (1.94, 5.08) |
| None/moderate alcohol consumption |  |  | 1.14 (0.98, 1.33) | 0.092 | / | / |
| Adequate fruit/vegetable consumption |  |  | 1.00 (0.87, 1.16) | 0.999 | / | / |

CHD means coronary heart disease. HTN means hypertension. DM means diabetes mellitus. ST means stroke, HR means hazard ratio, CI means confidence interval.

Physical inactivity, current smoking, excess alcohol consumption and inadequate fruit/vegetable consumption were used as reference groups. Models were adjusted for gender, race, body mass index, Townsend deprivation index and sedentary time, and use of antihypertensive medication, insulin, cholesterol lowering medication, and antiplatelet drugs.

**Supplementary Table 5.** Sensitivity analysis results when total amount of alcohol consumption was used

| Cardiometabolic multimorbidity | Number of  participants | Number of  deaths | HR (95% CI) | *P* | Increased life expectancy (95% CI) | |
| --- | --- | --- | --- | --- | --- | --- |
|  |  |  |  |  | Age at 45 years | Age at 65 years |
| **HTN+DM** |  |  |  |  |  |  |
| Regular physical activity | 5815 | 788 | 0.80 (0.68, 0.93) | 0.005 | 1.87 (0.50, 3.24) | 1.51 (0.41, 2.60) |
| Non-current smoking |  |  | 0.68 (0.55, 0.85) | 0.001 | 3.17 (1.20, 5.14) | 2.52 (0.97, 4.06) |
| None/moderate alcohol consumption |  |  | 0.91 (0.78, 1.05) | 0.183 | / | / |
| Adequate fruit/vegetable consumption |  |  | 0.99 (0.85, 1.17) | 0.949 | / | / |
| **HTN+CHD** |  |  |  |  |  |  |
| Regular physical activity | 7723 | 1126 | 0.76 (0.66, 0.88) | <0.001 | 2.05 (0.86, 3.23) | 1.74 (0.74, 2.74) |
| Non-current smoking |  |  | 0.40 (0.34, 0.46) | <0.001 | 7.10 (5.25, 8.95) | 5.87 (4.37, 7.37) |
| None/moderate alcohol consumption |  |  | 0.93 (0.82, 1.06) | 0.280 | / | / |
| Adequate fruit/vegetable consumption |  |  | 0.88 (0.77, 1.02) | 0.097 | / | / |
| **HTN+ST** |  |  |  |  |  |  |
| Regular physical activity | 2056 | 327 | 0.65 (0.50, 0.83) | 0.001 | 3.20 (1.00, 5.41) | 2.65 (0.82, 4.47) |
| Non-current smoking |  |  | 0.39 (0.29, 0.51) | <0.001 | 7.08 (3.94, 10.23) | 5.70 (3.14, 8.26) |
| None/moderate alcohol consumption |  |  | 0.81 (0.64, 1.02) | 0.069 | / | / |
| Adequate fruit/vegetable consumption |  |  | 0.87 (0.66, 1.14) | 0.302 | / | / |
| **HTN+DM+CHD** |  |  |  |  |  |  |
| Regular physical activity | 1571 | 399 | 0.77 (0.62, 0.96) | 0.020 | 2.55 (0.30, 4.80) | 2.00 (0.26, 3.74) |
| Non-current smoking |  |  | 0.61 (0.45, 0.84) | 0.002 | 4.75 (1.44, 8.05) | 3.67 (1.18, 6.16) |
| None/moderate alcohol consumption |  |  | 0.99 (0.81, 1.22) | 0.957 | / | / |
| Adequate fruit/vegetable consumption |  |  | 1.10 (0.89, 1.37) | 0.378 | / | / |

CHD means coronary heart disease. HTN means hypertension. DM means diabetes mellitus. ST means stroke, HR means hazard ratio, CI means confidence interval.

Physical inactivity, current smoking, excess alcohol consumption and inadequate fruit/vegetable consumption were used as reference groups. Models were adjusted for gender, race, body mass index, Townsend deprivation index and sedentary time, and use of antihypertensive medication, insulin, cholesterol lowering medication, and antiplatelet drugs.

**Supplementary Table 6.** Sensitivity analysis results when a more restricted definition of healthy diet was used

| Cardiometabolic multimorbidity | Number of  participants | Number of  deaths | HR (95% CI) | *P* | Increased life expectancy (95% CI) | |
| --- | --- | --- | --- | --- | --- | --- |
|  |  |  |  |  | Age at 45 years | Age at 65 years |
| **HTN+DM** |  |  |  |  |  |  |
| Regular physical activity | 10298 | 1433 | 0.78 (0.70, 0.87) | <0.001 | 2.04 (1.03, 3.06) | 1.64 (0.83, 2.45) |
| Non-current smoking |  |  | 0.63 (0.54, 0.74) | <0.001 | 3.84 (2.37, 5.31) | 3.03 (1.88, 4.18) |
| None/moderate alcohol consumption |  |  | 0.99 (0.89, 1.11) | 0.925 | / | / |
| Adequate fruit/vegetable consumption |  |  | 1.00 (0.89, 1.11) | 0.929 | / | / |
| **HTN+CHD** |  |  |  |  |  |  |
| Regular physical activity | 11175 | 1664 | 0.78 (0.69, 0.87) | <0.001 | 1.98 (1.01, 2.95) | 1.65 (0.85, 2.45) |
| Non-current smoking |  |  | 0.43 (0.37, 0.48) | <0.001 | 6.79 (5.29, 8.29) | 5.52 (4.32, 6.73) |
| None/moderate alcohol consumption |  |  | 0.99 (0.90, 1.10) | 0.911 | / | / |
| Adequate fruit/vegetable consumption |  |  | 0.82 (0.74, 0.90) | <0.001 | / | / |
| **HTN+ST** |  |  |  |  |  |  |
| Regular physical activity | 3050 | 484 | 0.67 (0.55, 0.82) | <0.001 | 3.06 (1.24, 4.88) | 2.54 (1.02, 4.05) |
| Non-current smoking |  |  | 0.42 (0.33, 0.53) | <0.001 | 6.85 (4.15, 9.54) | 5.56 (3.35, 7.78) |
| None/moderate alcohol consumption |  |  | 0.98 (0.82, 1.18) | 0.869 | / | / |
| Adequate fruit/vegetable consumption |  |  | 0.93 (0.77, 1.12) | 0.452 | / | / |
| **HTN+DM+CHD** |  |  |  |  |  |  |
| Regular physical activity | 2921 | 756 | 0.83 (0.71, 0.97) | 0.018 | 1.76 (0.24, 3.27) | 1.38 (0.20, 2.55) |
| Non-current smoking |  |  | 0.60 (0.49, 0.74) | <0.001 | 4.71 (2.53, 6.90) | 3.63 (1.97, 5.28) |
| None/moderate alcohol consumption |  |  | 1.10 (0.94, 1.30) | 0.234 | / | / |
| Adequate fruit/vegetable consumption |  |  | 0.97 (0.84, 1.13) | 0.733 | / | / |

CHD means coronary heart disease. HTN means hypertension. DM means diabetes mellitus. ST means stroke, HR means hazard ratio, CI means confidence interval.

Physical inactivity, current smoking, excess alcohol consumption and inadequate fruit/vegetable consumption were used as reference groups. Models were adjusted for gender, race, body mass index, Townsend deprivation index and sedentary time, and use of antihypertensive medication, insulin, cholesterol lowering medication, and antiplatelet drugs.

**Supplementary Table 7.** Sensitivity analysis results when newly diagnosed participants were excluded

| Cardiometabolic multimorbidity | Number of  participants | Number of  deaths | HR (95% CI) | *P* | Increased life expectancy (95% CI) | |
| --- | --- | --- | --- | --- | --- | --- |
|  |  |  |  |  | Age at 45 years | Age at 65 years |
| **HTN+DM** |  |  |  |  |  |  |
| Regular physical activity | 9362 | 1331 | 0.76 (0.68, 0.86) | <0.001 | 2.22 (1.16, 3.28) | 1.77 (0.93, 2.62) |
| Non-current smoking |  |  | 0.64 (0.54, 0.76) | <0.001 | 3.66 (2.13, 5.20) | 2.87 (1.68, 4.07) |
| None/moderate alcohol consumption |  |  | 0.98 (0.87, 1.11) | 0.769 | / | / |
| Adequate fruit/vegetable consumption |  |  | 1.05 (0.94, 1.18) | 0.394 | / | / |
| **HTN+CHD** |  |  |  |  |  |  |
| Regular physical activity | 10243 | 1542 | 0.79 (0.70, 0.89) | <0.001 | 1.86 (0.86, 2.87) | 1.56 (0.72, 2.40) |
| Non-current smoking |  |  | 0.43 (0.37, 0.49) | <0.001 | 6.81 (5.25, 8.38) | 5.56 (4.30, 6.83) |
| None/moderate alcohol consumption |  |  | 1.01 (0.91, 1.12) | 0.902 | / | / |
| Adequate fruit/vegetable consumption |  |  | 0.88 (0.78, 0.99) | 0.039 | / | / |
| **HTN+ST** |  |  |  |  |  |  |
| Regular physical activity | 2748 | 446 | 0.70 (0.57, 0.86) | 0.001 | 2.80 (1.00, 4.61) | 2.29 (0.83, 3.75) |
| Non-current smoking |  |  | 0.41 (0.32, 0.52) | <0.001 | 7.01 (4.33, 9.69) | 5.59 (3.50, 7.68) |
| None/moderate alcohol consumption |  |  | 1.05 (0.86, 1.27) | 0.650 | / | / |
| Adequate fruit/vegetable consumption |  |  | 0.97 (0.78, 1.20) | 0.758 | / | / |
| **HTN+DM+CHD** |  |  |  |  |  |  |
| Regular physical activity | 2607 | 675 | 0.84 (0.71, 1.00) | 0.045 | 1.55 (-0.02, 3.12) | 1.24 (-0.00, 2.49) |
| Non-current smoking |  |  | 0.56 (0.45, 0.69) | <0.001 | 5.27 (2.92, 7.63) | 4.14 (2.37, 5.90) |
| None/moderate alcohol consumption |  |  | 1.18 (0.99, 1.41) | 0.061 | / | / |
| Adequate fruit/vegetable consumption |  |  | 0.97 (0.82, 1.15) | 0.746 | / | / |

CHD means coronary heart disease. HTN means hypertension. DM means diabetes mellitus. ST means stroke, HR means hazard ratio, CI means confidence interval.

Physical inactivity, current smoking, excess alcohol consumption and inadequate fruit/vegetable consumption were used as reference groups.

Models were adjusted for gender, race, body mass index, Townsend deprivation index and sedentary time, and use of antihypertensive medication, insulin, cholesterol lowering medication, and antiplatelet drugs.

**Supplementary Table 8.** Sensitivity analysis results when participants with new-onset cardiometabolic diseases during follow-up were excluded

| Cardiometabolic multimorbidity | Number of  participants | Number of  deaths | HR (95% CI) | *P* | Increased life expectancy (95% CI) | |
| --- | --- | --- | --- | --- | --- | --- |
|  |  |  |  |  | Age at 45 years | Age at 65 years |
| **HTN+DM** |  |  |  |  |  |  |
| Regular physical activity | 7857 | 756 | 0.81 (0.69, 0.96) | 0.012 | 1.69 (0.33, 3.04) | 1.37 (0.28, 2.47) |
| Non-current smoking |  |  | 0.61 (0.49, 0.76) | <0.001 | 4.19 (2.21, 6.17) | 3.37 (1.81, 4.94) |
| None/moderate alcohol consumption |  |  | 1.04 (0.89, 1.22) | 0.644 | / | / |
| Adequate fruit/vegetable consumption |  |  | 1.07 (0.92, 1.25) | 0.379 | / | / |
| **HTN+CHD** |  |  |  |  |  |  |
| Regular physical activity | 8814 | 1102 | 0.77 (0.67, 0.89) | <0.001 | 2.07 (0.88, 3.26) | 1.75 (0.75, 2.74) |
| Non-current smoking |  |  | 0.38 (0.33, 0.44) | <0.001 | 8.13 (6.34, 9.91) | 6.77 (5.34, 8.20) |
| None/moderate alcohol consumption |  |  | 1.00 (0.88, 1.13) | 0.969 | / | / |
| Adequate fruit/vegetable consumption |  |  | 0.89 (0.77, 1.03) | 0.121 | / | / |
| **HTN+ST** |  |  |  |  |  |  |
| Regular physical activity | 2273 | 295 | 0.67 (0.52, 0.87) | 0.002 | 3.14 (1.00, 5.29) | 2.78 (0.90, 4.66) |
| Non-current smoking |  |  | 0.39 (0.29, 0.53) | <0.001 | 7.66 (4.52, 10.79) | 6.71 (4.02, 9.41) |
| None/moderate alcohol consumption |  |  | 1.06 (0.83, 1.34) | 0.650 | / | / |
| Adequate fruit/vegetable consumption |  |  | 0.85 (0.65, 1.12) | 0.250 | / | / |
| **HTN+DM+CHD** |  |  |  |  |  |  |
| Regular physical activity | 2482 | 584 | 0.86 (0.72, 1.03) | 0.100 | 1.43 (-0.31, 3.16) | 1.13 (-0.23, 2.50) |
| Non-current smoking |  |  | 0.55 (0.44, 0.70) | <0.001 | 5.60 (3.04, 8.16) | 4.37 (2.45, 6.29) |
| None/moderate alcohol consumption |  |  | 1.08 (0.90, 1.30) | 0.408 | / | / |
| Adequate fruit/vegetable consumption |  |  | 1.09 (0.91, 1.30) | 0.345 | / | / |

CHD means coronary heart disease. HTN means hypertension. DM means diabetes mellitus. ST means stroke, HR means hazard ratio, CI means confidence interval.

Physical inactivity, current smoking, excess alcohol consumption and inadequate fruit/vegetable consumption were used as reference groups.

Models were adjusted for gender, race, body mass index, Townsend deprivation index and sedentary time, and use of antihypertensive medication, insulin, cholesterol lowering medication, and antiplatelet drugs.
